# Supplementary material for: Whole blood stabilization for the microfluidic isolation and molecular characterization of circulating tumor cells
Source: Nat Commun. 2017 Nov 23;8:1733. doi: 10.1038/s41467-017-01705-y (PMC5700979; doi:10.1038/s41467-017-01705-y)
Supplement: Supplementary file 1 — Supplementary Information [file 41467_2017_1705_MOESM1_ESM.pdf]

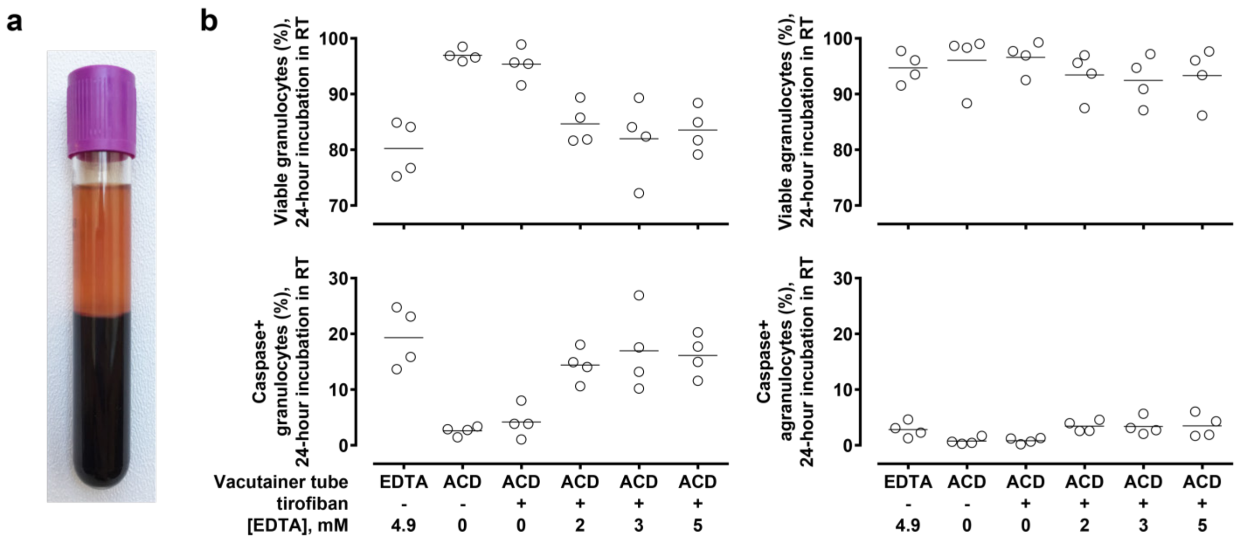

Supplementary Figure 1. Effects of EDTA and ACD anticoagulants on blood storage. (**a**) EDTA vacutainers (4.9 mM EDTA in 10 mL of blood) induce hemolysis after overnight storage in room temperature. (**b**) Viability of leukocytes quantified by imaging flow cytometry (as in Fig. 1). Blood was drawn into EDTA vacutainers, or ACD vacutainers with or without the addition of tirofiban (0.5 μg mL^{-1}) and/or EDTA (2-5 mM), and stored for 24 hours in room temperature (RT). All conditions that contained EDTA resulted in significantly lower granulocyte viability and higher rate of apoptosis than ACD or ACD with tirofiban ($p < 0.05$, pair-wise comparisons using one-way ANOVA with Tukey's posttest). Agranulocytes were insensitive to EDTA.

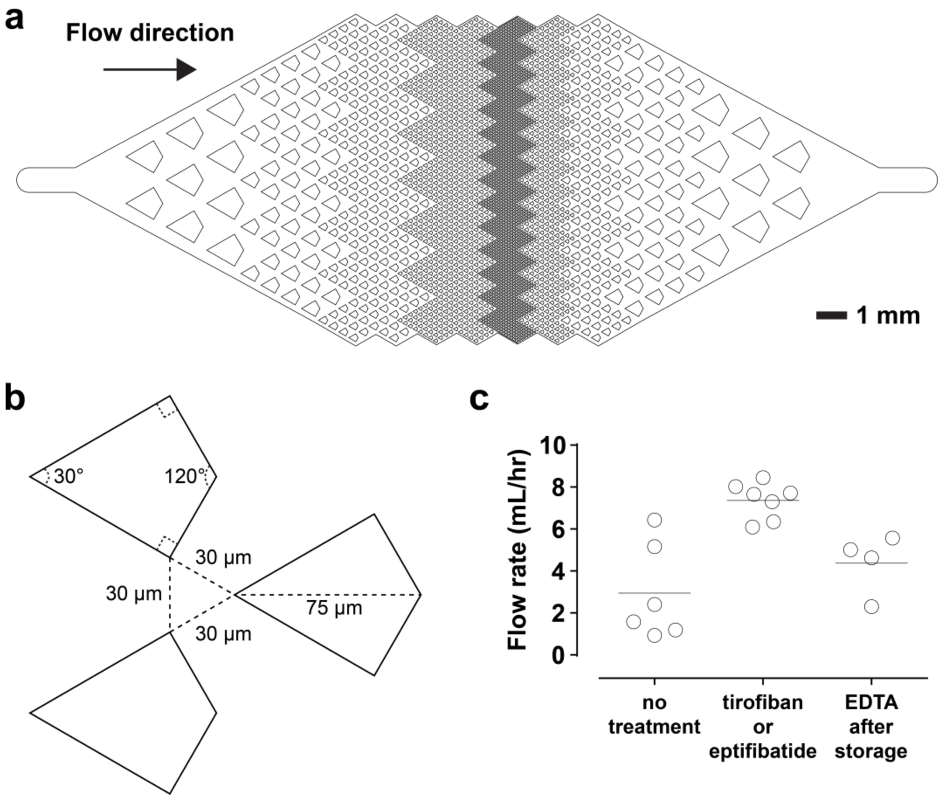

Supplementary Figure 2. Inhibition of glycoprotein IIb/IIIa is necessary and sufficient for clog-free microfluidic processing of stored blood. (**a**) A microfluidic filter device that contains kite-shaped microposts was used to assess clogging. The smallest features and gap sizes of this device is shown in (**b**). The height of the device is 50 µm. (**c**) Blood flow rate was used to quantify microfluidic clogging. ACD-anticoagulated blood was stored in 4 °C for 48-72 hours without any treatment, in the presence of tirofiban (0.5 µg mL^{-1}) or eptifibatide (20 µg mL^{-1}), or stored without treatment but supplemented with EDTA (4.7-5.7 mM final) after storage. The blood samples were then subjected to the same incubation protocol that mimics CTC-iChip processing conditions, and processed in this filter device under a constant pressure of 0.8 psi. Cold storage with tirofiban or eptifibatide enabled clog-free processing with consistent flow rate;

in comparison, no treatment ($p = 0.0007$) or EDTA supplementation post-storage ($p = 0.0276$) resulted in clogging and significantly decreased flow rates (one-way ANOVA followed by Tukey's posttest).

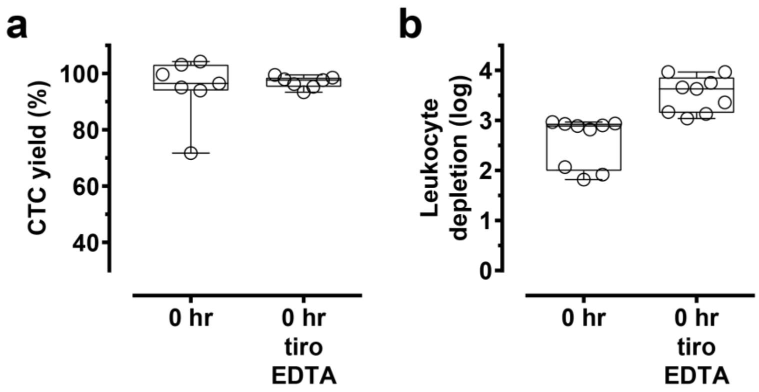

Supplementary Figure 3. Platelet stabilization greatly increases the purity of tumor cells isolated from fresh blood. (**a**) Yield of spiked tumor cells remained unchanged but (**b**) leukocyte depletion greatly increased with tiro-EDTA treatment ($p = 0.0003$, t test). Box-and-whiskers plots show median, interquartile range, maxima, and minima.

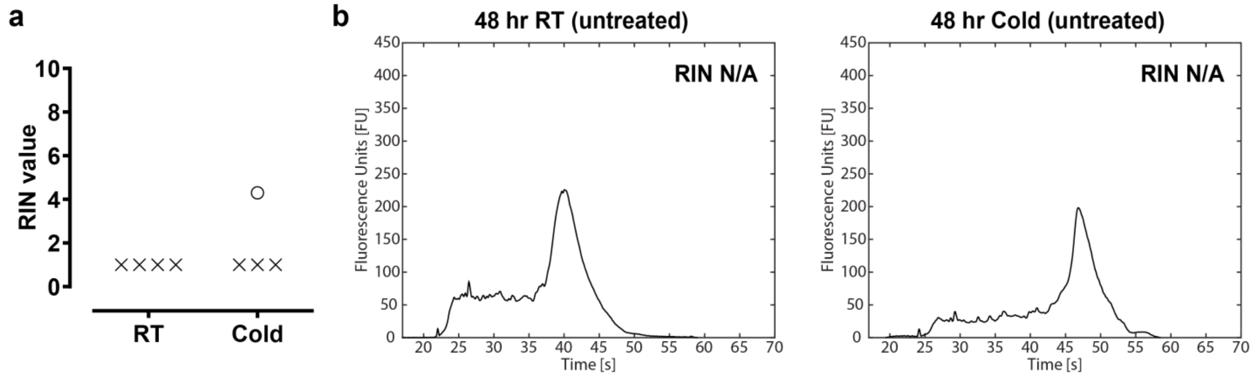

Supplementary Figure 4. RNA quality of patient CTCs is poor in the absence of tiro-EDTA treatment. Patient blood was stored in RT or cold for 48-72 hours without tirofiban and processed using the CTC-iChip. (**a**) The quality of the RNA was poor with only 1 out of 8 CTC products showing a computable RIN value (4.3; cold storage). For the rest of the CTC products, the RNA was so degraded (**b**) that RIN values were not available (N/A). We therefore assigned a RIN value of 1 (×; lowest possible RIN value) for easy visualization in (**a**). We attribute the poor RNA quality to processing failure of the CTC-iChip when tiro-EDTA is absent, due to microfluidic clogging and the resulting cellular damage, as well as the substantial increases in RBC and leukocyte contamination (Fig. 3).

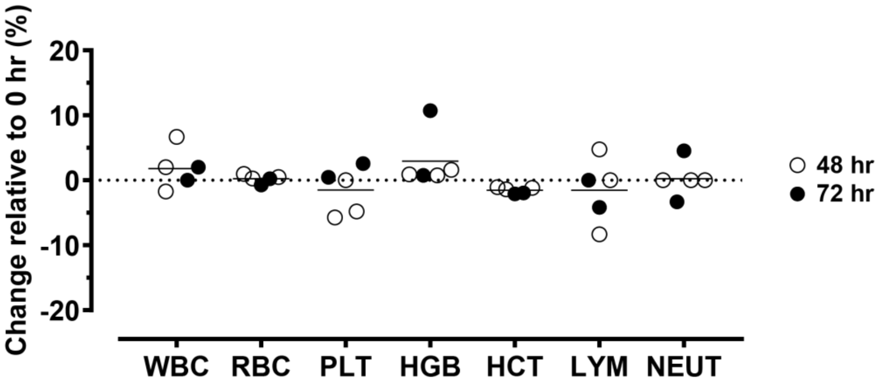

Supplementary Figure 5. Complete blood counts with white blood cell differential remain stable after cold preservation with tiro-EDTA treatment. Shown are percentage changes of the respective quantities relative to 0 hour. WBC, white blood cell count; RBC, red blood cell count; PLT, platelet count; HGB, hemoglobin concentration; HCT, hematocrit; LYM, lymphocyte count; NEUT, neutrophil count.

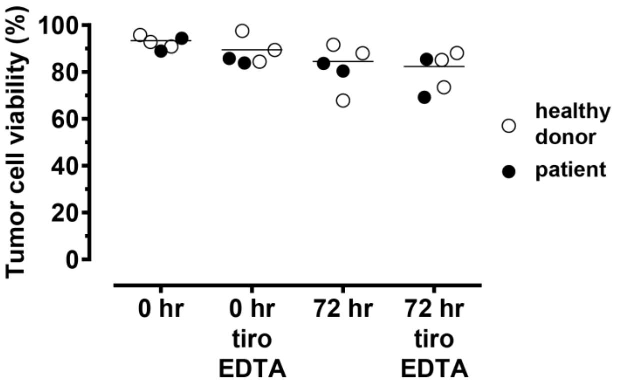

Supplementary Figure 6. Spiked tumor cells remain highly viable after hypothermic blood preservation. Shown are the percentages of viable (calcein+/caspase-) LNCaP cells in healthy donor or patient blood, analyzed immediately after spike or after 72 hours of hypothermic storage. The platelet stabilization treatment (tiro-EDTA) did not impact tumor cell viability.

Supplementary Table 1. Binary concordance of cancer- and prostate-specific gene detection between fresh and preserved blood.

	AR				ATP6V1G1				CNPY2				GUCY1A3				HSD17B4				KLK2				KLK3 (PSA)				MCCC2				NKX3.1				PDLIM				SOX4				AR-V7 (by ddPCR)				
**Patient No.**	**0**	**24**	**48**	**72**	**0**	**24**	**48**	**72**	**0**	**24**	**48**	**72**	**0**	**24**	**48**	**72**	**0**	**24**	**48**	**72**	**0**	**24**	**48**	**72**	**0**	**24**	**48**	**72**	**0**	**24**	**48**	**72**	**0**	**24**	**48**	**72**	**0**	**24**	**48**	**72**	**0**	**24**	**48**	**72**	**0**	**24**	**48**	**72**	**Concordance (%)**
Patient #1	1	1			1	1			1	1			1	1			1	1			1	1			1	1			1	1			1	1			1	1			1	1							100.0%
Patient #2.1	1	1			1	1			1	1			1	1			1	1			1	1			1	1			1	1			1	1			1	1			1	1			1	1			100.0%
Patient #3.1	1	1			1	1			1	1			1	1			1	1			1	1			1	1			1	1			1	1			1	1			1	1							100.0%
Patient #3.2	1		1		1		1		1		0		1		1		1		1		1		1		1		1		1		1		1		0		1		1		1		1						81.8%
Patient #3.3	0			1	1			1	0			1	1			1	1			1	0			1	0			1	1			1	0			0	1			1	1			1					63.6%
Patient #4	1		1		1		1		1		1		1	1			1	1			1		1		1		1		1		1		1		1		1		1		1		1						100.0%
Patient #2.2																																													1	1			
Patient #5																																													1	1			
Patient #6.1																																													1		1		
Patient #6.2																																													1		1		
Patient #7																																													0		0		
Patient #8																																													0		0		
Patient #9																																													0		0		
Patient #10																																													1			1	
																																																**Overall concordance**	**92.0%**

Shown are the top 11 genes with the most stringent prostate-cancer specificity (per the GTEx Portal) detected by RNA-Seq, and *AR-V7* detected using ddPCR. Genes that were detected in each experimental condition are assigned a value of "1", while genes that were not detected are assigned a value of "0." A concordant result is such that, for that particular gene, both the 0 hour and preserved blood share the same number notation. Binary concordance is calculated within each patient and across all patients. An overall binary concordance of 92.0% reflects the concordance across 14 blood draws and 12 genes.

Supplementary Table 2. Amplicon sizes and sequences of forward and reverse primers for single-cell RT-qPCR used in the Fluidigm platform.

Gene	Category	Amplicon Size	Forward primer	Reverse primer
KLK3 (PSA)	AR-on	71	CACCTGCTCGGGTGATTCTG	TGCCCCATGACGTGATACCT
SORD	AR-on	95	ACCACCGTACCCCTACTGC	AGCATCGAAATCGCCACT
NKX3.1	AR-on	91	AGAACGACCAGCTGAGCAC	TAAGACCCCAAGTGCCTTTC
FKBP5	AR-on	69	GGATATACGCCAACATGTTCAA	CCATTGCTTTATTGGCCTCT
ACSL3	AR-on	71	TCAGGGATACGGGCTCACT	TGCCAGTATTGTAGTCCCACAC
DBI	AR-on	92	CAGAGGAGGTTAGGCACCTTA	TATGTCGCCCACAGTTGCTTG
KLK2	AR-on	82	CTGCCCATTGCCTAAAGAAG	ACCCTCTGGCCTGTGTCTT
BRP44	AR-on	75	TTATCAGTGGGCGGATGACAT	GCTGTACCTTGTAGGCAAATCTC
ABHD2	AR-on	74	GCCCAACATTGAATTGACCT	AGTTCACCATGGCTCCAAAT
KRT8	AR-on	91	AGGGCTGACCGACGAGAT	CACCACAGATGTGTCCGAGA
SELENBP1	AR-off	95	ACCCAGGGAAGAGATCGTCTA	ACTTGGGGTCAACATCCACAG
CAMK2N1	AR-off	92	GACACCAACAACTTCTTCGGC	TCATCTTCAATAACAACCCGCTT
PEG3	AR-off	124	CACGCAGTTCCAAATCGGGA	CGCTGCTGGATCACTGACTC
FOLH1 (PSMA)	AR-off	75	CCATTAGGGTTACCAGACAGGC	CCCTGCATACTTGTTGTGGC
NIPSNAP3A	AR-off	96	CAGAGTACGGAGCACTCAACAG	TGGGATCCTCATGGGACTTA
SOX4	AR-off	107	GACCTGCTCGACCTGAACC	CCGGGCTCGAAGTTAAAATCC
SEMA6A	AR-off	119	ACATTGCTGCTAGGGACCATA	TCTGCATGTGTCTACATCGGC
KRT18	epithelial	86	GTTGACCGTGGAGGTAGATGC	GAGCCAGCTCGTCATATTGGG
TACSTD1 (EpCAM)	epithelial	111	CCATGTGCTGGTGTGTGAA	TGTGTTTTAGTTCAATGATGATCCA
FN1	mesenchymal	86	GAAGCCGAGGTTTTAACTGC	ACCCACTCGGTAAGTGTTCC
AR	prostate	103	GCCTTGCTCTCTAGCCTCAA	GGTCGTCCACGTGTAAGTTG
MYC	stem cell	73	GCTGCTTAGACGCTGGATTT	TAACGTTGAGGGGCATCG
BIRC5	proliferation	88	GCCCAGTGTTTCTTCTGCTT	AACCGGACGAATGCTTTTTA
MYBL2	proliferation	116	CTTGAGCGAGTCCAAAGACTG	AGTTGGTCAGAAGACTTCCCT
CCNB1	proliferation	103	ACATGGTGCACTTTCCTCCT	AGGTAATGTTGTAGAGTTGGTGTCC
CCND1	proliferation	75	TGGAGCCCGTGAAAAAGAGC	TCTCCTTCATCTTAGAGGCCAC
PTPRC (CD45)	WBC	81	AGTCAAAGTTATTGTTATGCTGACAGA	TGCTTTCCTTCTCCCCAGTA
CD34	WBC	67	GCGCTTTGCTTGCTGAGT	GGGTAGCAGTACCGTTGTTGT
FCGR3B (CD16)	WBC	107	GGGCTTGTTGGGAGTAAAAA	ACTTGGTACCCAGGTGGAGA
CD14	WBC	92	TTAGGCTCCCGAGTCAACAG	CTGCAGGGCATCTAGGGTTC
FUT4 (CD15)	WBC	139	CCGCTACTACCACCAACTGAG	CTGCGAGTTCTCGAAAGCCA
GAPDH	housekeeping	101	ACAACTTTGGTATCGTGGAAGG	GCCATCACGCCACAGTTTC
ACTB (ActB2)	housekeeping	120	AGGCCAACCGCGAGAAG	ACAGCCTGGATAGCAACGTACAT
RPLP0	housekeeping	97	CAGATTGGCTACCCAACTGTT	GGAAGGTGTAATCCGTCTCCAC

Genes are grouped into categories (i.e., AR-on, AR-off, epithelial, mesenchymal, prostate, stem cell, proliferation, and housekeeping). Also included are leukocyte markers for exclusion.
